# The neuropeptide Y single-nucleotide polymorphism *rs16147:T*>*C* moderates the effect of alcohol dependence on depression in male Chinese Han population

**DOI:** 10.3389/fpsyt.2022.1012850

**Published:** 2022-09-29

**Authors:** Xiaojie Wei, Fangfang Cai, Siyao Zhou, Jinjing Zhang, Kewei Xu, Guanghui Shen, Huankun Sun, Fan Yang, Liuzhi Hong, Yang Zou, Yu-Hsin Chen, Yanlong Liu, Li Chen, Fan Wang, Wei Wang

**Affiliations:** ^1^Affiliated Cixi Hospital, Wenzhou Medical University, Ningbo, China; ^2^School of Mental Health, Wenzhou Medical University, Wenzhou, China; ^3^The Affiliated Kangning Hospital, Wenzhou Medical University, Wenzhou, China; ^4^Department of Psychology, College of Liberal Arts, Wenzhou-Kean University, Wenzhou, China; ^5^Beijing Hui-Long-Guan Hospital, Peking University, Beijing, China; ^6^Zhejiang Provincial Clinical Research Center for Mental Disorders, The Affiliated Wenzhou Kangning Hospital, Wenzhou Medical University, Wenzhou, China

**Keywords:** NPY *rs16147*:*T*>*C*, alcohol use disorder, depressive disorder, interaction, SNP

## Abstract

**Background:**

Previous studies suggest that alcohol dependence is associated with depression, however, the effect of alcohol dependence varies from individual to individual, which may be due to different genetic backgrounds. The interactions between alcohol dependence and different gene polymorphisms may finally shape the onset of depression. Neuropeptide Y (NPY), which can maintain homeostasis from high-stress stimulation, may protect individuals from the onset of depression. Here, we explored whether the NPY *rs16147:T*>*C* has an association with depression in individuals with alcohol dependence during the period of alcohol dependence withdrawal.

**Methods:**

A total of 455 males with alcohol dependence were recruited. The scale of Michigan Alcoholism Screening Test (MAST) and Self-Depression Scale (SDS) were respectively used to analyze the condition of alcohol dependence and depression. Genomic DNA was extracted from each blood sample and NPY polymorphisms were genotyped. The interaction between NPY *rs16147:T*>*C* and alcohol dependence on depression was first analyzed. Then, region of significance analysis was used to confirm which model provided the best fit for the interaction (diathesis-stress or differential susceptibility). Finally, by using internal replication analyses, the accuracy and robustness of the interaction results were improved.

**Results:**

Alcohol dependence was positively correlated with depression. CC homozygotes of NPY *rs16147:T*>*C* exhibited less depression when exposed to low alcohol dependence, but more depression when exposed to high alcohol dependence. Individuals with the T allele showed the opposite result.

**Conclusion:**

NPY *rs16147:T*>*C* might be correlated with susceptibility for depression in males during alcohol dependence withdrawal. The findings support the differential susceptibility model.

## Introduction

Alcohol use is an established facet of diverse cultural contexts and religions, providing perceived pleasure to many users and facilitating social interaction. However, excessive alcohol use is also a principal causal factor in a number of mental health conditions, including alcohol dependence and other alcohol use disorders (1). According to the WHO global status report on alcohol and health in 2018, alcohol dependence (identified as an impairment of self-control over his or her drinking) is often associated with mental health problems such as depression (2). Further studies have shown that alcohol dependence significantly increases the risk of suffering from depression (3, 4). Alcohol use disorder is characterized by repeated withdrawals, followed by relapses, resumption of drinking. Alcohol dependent withdrawal (ADW) is caused by a cessation of long-term ethanol consumption and is characterized by symptoms of hyperactivity of the central nervous system, hyperactivity of the autonomic nervous system, causing physical and emotional disorders (5). ADW-related mood disorders, including depression, anxiety, irritability, are a significant cause of alcohol dependence relapse (6–8). However, reports on the effect of ADW and the onset and emergence of depression varies greatly across individuals (9).

The literature on alcohol dependence withdrawal indicates that not all individuals experience symptoms of depression in the context of ADW. Several depression studies indicate that more than 50% of depression patients have experienced severe adversity before the onset of the illness, while some people facing severe stress never have symptoms of depression (10, 11). For example, Caspi et al. found subjects with short allele of 5-HTTLPR (homozygotes or heterozygotes) exhibited more depressive symptoms and had more possibility to diagnose as depression than individuals with long allele homozygotes of 5-HTTLPR when exposed to life stress (12). To further investigate factors that lead to differences in the development of depression on an individual level, depression and alcoholism studies began to investigate the association of polymorphisms in various genetic regions of alcohol dependent subjects (13–15). Amongst cyclic AMP (cAMP), protein kinase A, cAMP responsive element-binding, and neuropeptide Y (NPY), the role of NPY in the development of depression within the context of alcohol dependence is well-established (13, 15–17). NPY, a highly conserved 36-residue peptide, is reported to be densely distributed in amygdala, hippocampus, neocortex and other areas related to psychopathology, playing a pivotal role in regulating emotion response (18). Many studies have shown that the expression level of NPY in the brain is closely related to the occurrence of depression. For example, people with low levels of NPY in the brain have a higher risk of depression (19). Conversely, administration of NPY have anti-stress effects, and thereby can reduce emotional responses on depression (20).

Single nucleotide polymorphisms (SNPs) are genetic variants of one nucleotide and these changes could have functional implications. On the one hand, SNPs can influence rates of transcription causing changes in the production of encoded protein when present in regulatory sites of a gene. On the other hand, in the coding regions, SNPs can alter protein structure and hence function, resulting in the development of disease or change in response to a drug or environmental toxin (21). Thus, researchers have used SNPs as biomarkers in many disease genetics and pharmacogenomic studies. Located on chromosome 7p15.3, the human NPY gene has four exons, of which *rs16147* is the main genetic variant described in this gene and it is located within the promoter region upstream of the NPY gene (22). Previous studies have identified NPY *rs16147:T*>*C*, a single promoter variant, responsible for the expression of NPY (15). Furthermore, it reported that NPY *rs16147:T*>*C* genotypes were associated with stress experiences, which can predict stress-related outcomes. In animal models, NPY reduces stress effects (20, 23) and alcohol consumption (24), and opposes the actions of corticotropin-releasing factor (CRF) (25) as well as responsible for the anxiogenic and stress-like consequences of alcohol dependence withdrawal (16).

Yet to date, few studies have examined the exact form of the interaction between the environment and NPY *rs16147:T*>*C* gene polymorphism. According to the literature on gene-by-environment (G × E) interactions, the interaction between environment and gene polymorphism can be fitted into two model. The *diathesis-stress* model of environmental action (26) suggests individuals with a risk gene are affected negatively by poor environments, whereas individuals with a different version of the same gene are relatively unaffected by environments. Where given the best environments, individuals with differing polymorphisms may exhibit similar levels of behavior, but behavior of the groups diverges with worsening environmental conditions. The *differential-susceptibility* model (27) on the other hand, suggests that individuals carrying “risk alleles” may simply be more malleable to changes in the environment. Wherein, individuals with a putative high-risk allele should exhibit poorer outcomes in poor environments and similar outcomes to individuals with a low-risk allele in average environments. However, in very good environments, individuals with a putative high-risk allele will show outcomes that are superior to individuals with the low-risk allele. In other words, individuals with “risk alleles” are malleable to environment conditions, benefitting from supportive environments but exhibiting poorer outcomes in poor environments.

The present study aims to understand the role of NPY *rs16147:T*>*C* on depression in patients with acute alcohol dependence withdrawal. According to the literature on alcohol dependence and depression, NPY *rs16147:T*>*C* is key to understanding the development of depression in alcohol dependence withdrawal population (28). Yet to date, the exact form of the interaction between the environment and NPY *rs16147:T*>*C* gene polymorphism remains unclear. To further understand the role of NPY *rs16147:T*>*C*, blood samples and scores of Michigan Alcoholism Screening Test (MAST), self-depression scale (SDS) were obtained from alcohol dependence patients from seven psychiatric hospitals. Confirmatory analytic approaches commonly used in previous studies were then conducted to identify the form of G × E interaction. Specifically to identify whether the role of NPY *rs16147:T*>*C* conforms to the *diathesis-stress* model or the *differential-susceptibility* model. Based on the current literature suggesting that T alleles of *rs16147:T*>*C* are protective under high stress conditions (15). It could be hypothesized that NPY *rs16147:T*>*C* may accord with the differential-susceptibility model, wherein NPY *rs16147:T*>*C* under very good environment conditions may show outcomes that are superior to individuals with the low-risk allele; but poorer outcomes in poor environments and similar outcomes in average environments compared to low-risk allele.

## Materials and methods

### Participants

A total of 455 males were recruited from seven psychiatric hospitals in northern China: Beijing Hui Long Guan Hospital, Shandong Mental Health Center, the Sixth Hospital in Changchun, Shenyang Mental Health Center, the Third Hospital in Inner Mongolia Autonomous Region, Hulunbuir Mental Health Center, and Tongliao Mental Health Center. All the patients recruited in the study met the criteria for alcohol dependence based on the Structured Clinical Interview for DSM-IV Axis I disorders.

The inclusion criteria were as follows: (1) diagnosis of alcohol dependence according to the DSM-IV; (2) male gender; (3) Han ethnicity; (4) provided written informed consent. The exclusion criteria were as follows: (1) presence of other substance abuse or dependence; (2) presence of severe cardiovascular disease, liver disease, or kidney disease; (3) participant, or a first-degree relative of the participant, has a serious mental illness; (4) individual declined to participate.

The participants were asked to complete a series of questionnaires and provide a blood sample for DNA extraction. All staff involved in this study were trained before the study commenced.

### Measures

#### Alcohol dependence

Alcohol dependence was assessed using MAST (13). The MAST is a self-report questionnaire comprising 25 items rated on a scale from 1 to 4, with a higher number corresponding to greater alcohol dependence. The scale has high internal consistency reliability, with an alpha value of 0.90 (15).

#### Depression

SDS was used to assess each participant's level of depression. The SDS contains 20 items that are rated on a scale from 1 to 4, with higher numbers corresponding to more frequent symptoms. The higher the total score, the more severe the depressive symptoms (17). Considering that the ADW patients are able to cooperate in answering the questionnaire about 20 days later, SDS is used to measure the severity of depressive symptoms 3 weeks after hospitalization.

### Genotyping

Genomic DNA was extracted from each blood sample using standard techniques. The NPY *rs16147:T*>*C* was conducted using the TaqMan SNP genotyping assay (cat number: 4351374; ABI: Applied Biosystems Inc., Foster City, CA, USA). The probes of SNPs were analyzed from ABI assay on demand kit.

### Statistical analysis

First, the Hardy–Weinberg equilibrium for genotype distributions of NPY *rs16147:T*>*C* was tested using the c2 test for goodness of fit. Then, Pearson correlations were examined between genetic polymorphisms, age, years of education, alcohol dependence, and depression. Consistent with other research, CT and TT genotypes were collapsed into a T-allele group and coded as 1; the CC genotype was coded as 0. Then, traditional linear regression was used to test the interaction between alcohol dependence and the *rs16147:T*>*C* polymorphism. When a significant interaction was found, region of significance (RoS) analysis was used to examine the form of the interaction (29).

Finally, a re-parameterized regression model was fitted to examine the specific pattern of gene × environment pattern (30), which had the form:


$$\text{Y}=\{\begin{array}{c} \text{Group}:\text{D}=0\;B_{0}+B_{1}\;(\text{X}-\text{C})+B_{3}{\text{X}}_{2}+B_{4}{\text{X}}_{3}+E\\ \text{Group}:\text{D}=1\;B_{0}+B_{2}\;(\text{X}-\text{C})+B_{3}{\text{X}}_{2}+B_{4}{\text{X}}_{3}+E \end{array}$$

where Group is the allelic group, X is alcohol dependence, X_2_ and X_3_ are the demographic covariates age and years of education, Y is the dependent variable of depression, and C is the crossover point where the slopes of the different groups cross. What distinguishes the diathesis-stress model and differential susceptibility model is the estimate and interval estimate of crossover point C. If the estimate and interval estimate of crossover point C fall within the range of alcohol dependence, the model is consistent with the differential susceptibility model. Otherwise, if crossover point C is over the maximum of alcohol dependence, the model is consistent with the diathesis-stress model.

The diathesis-stress model and differential susceptibility model can be further subdivided into “strong” and “weak” versions. Strong versions assume that “non-risk/non-plasticity allele” carriers are not susceptible to the environment. Weak versions assume that both allele carriers are susceptible to the environment but “non-risk/non-plasticity allele” carriers are less susceptible to the environment than “risk/plasticity allele” carriers. These models are nested within each other. Thus, the F test was used to compare the models and identify a difference in the parameter estimates. For non-nested models, the Akaike information criterion (AIC) and Bayesian Information Criterion (BIC) were compared to evaluate which model was a better fit.

All *p*-value of <0.05 were considered as statistically significant. IBM SPSS Statistics 22.0 (IBM SPSS, Inc.) and R-language (R Foundation for Statistical Computing, Vienna, Austria) were used for the statistical analyses.

## Results

### Descriptive statistics

Table 1 showed the demographic and clinical characteristics of the participants. The mean age of the participants was 44.31 years (SD = 9.29). Regarding their education, the average number of years in formal schooling was 10.69 (SD = 2.84). And the mean scores of MAST was 9.28 (SD = 5.47) and SDS was 56.06 (SD = 11.39). The condition of marital status and living patterns were also listed in the table. Besides, of the 455 male inpatients, 60 (13.19%) were CC homozygotes, 213 (46.81%) were CT heterozygotes, and 182 (40.00%) were TT homozygotes. The genotype distribution of NPY *rs16147:T*>*C* was consistent with the Hardy–Weinberg equilibrium (χ2 = 0.04, *p* > 0.05, Supplementary Table 1). A series of *t*-tests were then conducted to examine whether male inpatients with and without alcohol dependence and depressive symptoms differed in terms of the polymorphism NPY *rs16147:T*>*C*. The results revealed no significant differences (alcohol dependence: *t* = 1.13; depression: *t* = 0.11, both *p* > 0.05, Supplementary Table 2).

**Table 1:** Demographic and clinical characteristics of the study subjects (*N* = 455).

**Factors**	**Mean (SD) or *N* (%)**
Age (years)	44.31 (9.29)
Education years (years)	10.69 (2.84)
**Marital status (** ***n*** **)**
Married	328 (72.1%)
Not married	51 (11.2%)
Divorced	70 (15.4%)
Widowed	6 (1.3%)
**Living patterns (** ***n*** **)**
Live with family	361 (79.3%)
Living with non-family	94 (20.7%)
MAST (scores)	9.28 (5.47)
SDS (scores)	56.06 (11.39)

MAST, the Michigan Alcoholism Screening Test; SDS, the Self-rating Depression Scale.

### Correlation of MAST and SDS scores

The descriptive statistics for each research variable are shown in Table 2. Male inpatients' MAST scores were positively correlated with SDS scores (*r* = 0.24, *p* < 0.01). Whilst MAST scores were negatively correlated with the educational years (*r* = −0.2, *p* < 0.01). No significant correlation between polymorphisms of NPY *rs16147:T*>*C* and MAST (*p* > 0.05), as well as SDS scores (*p* > 0.05) were observed.

**Table 2:** Descriptive statistics and correlations among study variables.

	**NPY *rs16147:T>C***	**Age**	**Educational years**	**Alcohol dependence**	**Depression**
NPY *rs16147:T>C*	1				
Age	0.01	1			
Educational years	−0.02	−0.38**	1		
Alcohol dependence	−0.05	0.15**	−0.20***	1	
Depression	−0.01	−0.01	−0.03	0.24***	1
*M*	(–)	44.31	10.69	9.28	56.06
*SD*	(–)	9.29	2.84	5.47	11.39

NPY, neuropeptide Y; M, mean; SD, standard deviation. **p < 0.01; ***p < 0.001.

### Effect of interactions between NPY genotyping and alcohol dependence on depression

**Figure 1:**
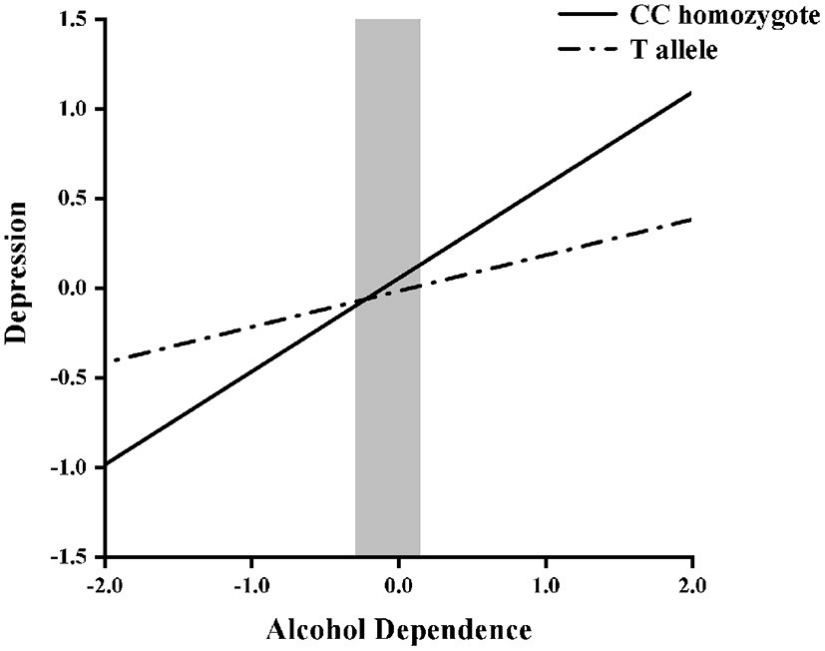
Region of significance test on depression from alcohol dependence in NPY *rs16147:T*>*C* allelic groups. Gray shaded area represents 95% CI of the crossover point C of the interaction on the alcohol dependence axis. 95% CI of C ranged from −0.29 to 0.15. T allele carriers: CT or TT.

Next, hierarchical regression models were used to predict depression from alcohol dependence for different allelic groups, with age and years of education as covariates. With regard to the main effects, Table 3 shows that alcohol dependence was positively related to depressive symptoms (β = 0.52, *p* < 0.05), but that there was no main effect of genotype (β = −0.02, *p* = 0.65). Furthermore, the interaction between NPY *rs16147:T*>*C* and alcohol dependence was significant (β = −0.29, *p* < 0.05). Then, the RoS test was used to examine the interaction effect. As shown in Figure 1, the slopes for alcohol dependence on depression were as follows: CC homozygotes, β = 0.25, *t* = 16.44, *p* < 0.01; T allele carriers, β = 0.23, *t* = 4.90, *p* < 0.01. Relative to T allele carriers, CC homozygotes were more likely to be affected by alcohol dependence causing depression.

**Table 3:** Interaction between NPY *rs16147:T*>*C* and alcohol dependence on depression during acute alcohol dependence withdrawal.

**Variables**	**Depression**					
	***ΔR^2^***	***B* (SE)**	**β**	***t***	***p***	**95% confidence interval**
Age	0.01	0.01 (0.01)	0.05	0.92	0.36	−0.01to 0.02
Educational years		−0.00 (0.02)	−0.01	−0.11	0.91	−0.04 to 0.03
Alcohol dependence	0.05	0.52 (0.12)	0.52	4.29	< 0.001***	0.28 to 0.76
NPY *rs16147:T>C*		−0.06 (0.14)	−0.02	−0.45	0.65	−0.33 to 0.21
Alcohol dependence × NPY*rs16147:T>C*	0.01	−0.32 (0.13)	−0.29	−2.41	0.02*	−0.57 to−0.06

NPY, neuropeptide Y; SE, standard error. *p < 0.05; ***p < 0.001.

### Internal replication analyses

In order to improve the accuracy and robustness of the interaction results, re-parameterized regression model was fitted to examine the specific pattern of gene × environment. The fit of the weak differential susceptibility model, Model B, yielded a significant *R*^2^ = 0.07, *p* < 0.001 (Table 4), explained a significant amount of variance in depression, in which the slope for T allele group was significant (*B*_2_ = 0.52, *p* < 0.001). The estimated crossover point C and 95% CI of C both fell within the range of alcohol dependence, *C* = −0.19 (SE = 0.43), 95% CI = [−1.03, 0.65]. Thus, Model B provides strong support for the weak differential susceptibility model, indicating that the T allele carriers with alcohol dependence is less susceptible to depression than CC homozygotes.

**Table 4:** Results for re-parameterized regression model for depression.

	**Differential susceptibility**		**Diathesis-stress**	
**Parameter**	**Strong:**	**Weak:**	**Strong:**	**Weak:**
	**Model A**	**Model B**	**Mode C**	**Mode D**
*B_0_*	−0.21 (0.36)	−0.24 (0.37)	−0.22 (0.36)	0.15 (0.36)
*B_1_*	0.00 (–)	0.21*** (0.05)	0.00 (–)	0.24*** (0.05)
*C*	−0.10 (0.27)	−0.19 (0.43)	1.51 (–)	1.51 (–)
*95%CI of C*	[−0.63, 0.43]	[−1.03, 0.65]	(–)	(–)
*B_2_*	0.51*** (0.12)	0.52*** (0.12)	0.17*** (0.07)	0.32*** (0.07)
*B_3_*	0.01 (0.01)	0.01 (0.01)	0.01 (0.01)	0.01 (0.01)
*B_4_*	0.01 (0.02)	−0.01 (0.02)	0.01 (0.02)	−0.00 (0.02)
*R^2^*	0.04	0.07	0.01	0.06
*F* (*df*)	4.48*** (4,450)	8.90*** (5,449)	2.11 (3,451)	7.68*** (4,450)
*F vs. A(df)*	(–)	16.98***	11.41***	(–)
*F vs.B(df)*	16.98***	(–)	14.40***	4.56*
AIC	1284.47	1269.58	1293.87	1272.19
BIC	1309.19	1298.42	1314.47	1296.91

AIC, Akaike information criterion; BIC, Bayesian information criterion. *p < 0.05; ***p < 0.001.

## Discussion

Alcohol dependence withdrawal can be regarded as an acute stressor, and NPY is conducive to coping with stress-related psychological disorders. Thus, the purpose of this study was to explore the interaction between alcohol dependence and NPY *rs16147:T*>*C* and its potential additional meaning in the occurrence of depression during the period of ADW. The results revealed that during the period of ADW, when the degree of alcohol dependence was low, NPY *rs16147:T*>*C* CC homozygotes showed a lower degree of depression compared with those who carried the NPY *rs16147:T*>*C* T allele. When the degree of alcohol dependence was high, the result was reversed. Further, the model conformed to the differential susceptibility model.

We firstly identified the relevance between the NPY *rs16147:T*>*C* × alcohol dependence and depression, then competitive model-testing analysis was employed to evaluate which gene and disease interaction model was the best fit for the data. The results revealed that the weak differential susceptibility model was the best fit. This indicates that CC homozygotes react differently to depression in the adverse or positive condition of alcohol dependence during the period of ADW. Individual genetic variation and the interactions between genes and external factors may characterize neural circuits and neurochemical functions, which represent the psychological strength of adaptable individuals. Stress-related events (such as ADW) can increase an individual's susceptibility to serious psychiatric problems such as depression. Considering that genetic factors contribute to recovery, it is necessary to identify candidate genetic variations to explain genetic patterns.

Alcohol dependence withdrawal can give rise to increased CRF synthesis and release (31). CRF is a stress-promoting neuropeptide which is dysregulated by long-term high-dose alcohol exposure. It can induce anxiogenic behaviors or other stress-related symptoms (25). Interestingly, NPY and CRF show high neuroanatomical overlap but opposite function (32). The current researches have consistently pointed out that NPY mainly counteracts alcoholism anxiety and stress-related symptoms caused by CRF in alcohol dependence (25), which suggests the effect of NPY on emotional homeostasis. And there is evidence that NPY may, through the GABAA receptor, significantly counteract the central CRF and attenuate the biological actions of CRF, in turn weaken stress responses (33).

The expression of NPY is related to the polymorphism of the NPY gene as well as change in NPY expression. For the NPY gene, one of the main genetic variations is *rs16147:T*>*C*, which is located in the promoter region upstream of the NPY gene (34). The results of this study suggest that *rs16147:T*>*C* may have a connection in the regulation of emotions, especially in terms of an interaction with alcohol dependence. In relation to the underlying internal mechanisms, there are still some unknowns. Even though, existing researches show that CC homozygotes is associated with stress-related psychopathology (35); thus, the current findings may be explained by the fact that individuals with genetic variation respond differently to different degrees of alcohol dependence. Zhou et al. found that healthy individuals with low NPY expression genotypes and CC homozygotes had increased amygdala activity during the stress response (36). Neuroimaging studies of depression have suggested a diverse array of brain regions, including the amygdala, ventral striatum, thalamus, and cingulate. Among the regions associated with depression, the amygdala has received some of the most intense empirical scrutiny. Meanwhile, studies have led many to conclude that amygdala hyper-reactivity increases the risk for depression and other, often co-occurring internalizing illnesses (37). Therefore, we can presume that depression is associated with an increased response to negative stimuli in the amygdala. When the degree of alcohol dependence is increased, the amygdala activity in CC homozygotes may be enhanced. This may explain why CC homozygotes are more likely to be depressed in an adverse environment. Additionally, the current results indicated that individuals with the T allele exhibited about the same amount of depression in the case of severe alcohol dependence and in the condition of mild alcohol dependence. This finding aroused our interest. It has been reported that NPY *rs16147:T*>*C* can interact with chronic stress and affect autonomic control, of which the T genotype is associated with vagal activity. Under high chronic stress, vagus nerve activity was found to be increased in patients with the T genotype (38). Thus, it may be that with an increased degree of alcohol dependence, T allele carriers can increase their capacity to cope with depression by regulating the levels of vagal activity.

These findings shed light on the potential mechanism of NPY *rs16147:T*>*C* in depression during ADW. According to the mechanism of genetic susceptibility, individuals of different genotypes showed different characteristics under stress. The early adverse environment may change the concentration of NPY, which can further induce stress-related psychopathology for some genotypes. Moreover, for some individuals, genetic resilience may enable the generation of more psychological resources in the face of adverse environmental stressors. In the current study, T allele carriers were able to adjust their state of emotion when dealing with adverse alcohol dependence, while CC homozygotes may have trouble dealing with a bad mood when experiencing severe alcohol dependence.

These findings contribute to the existing literature by providing valuable information about the latent etiology of alcohol dependence and depression, which also have some practical significance. The SNP (NPY *rs16147:T*>*C*) might be one candidate biomarker for screening individuals with a higher risk of developing depression in ADW. For ADW patients with CC homozygotes, public-health workers are supposed to heighten their vigilance in whether they are accompanied by higher depressive tendency during acute withdraw in hospital. Improving the disease detection rate is helpful for early prevention and treatment.

Several limitations of this study should be addressed. First, this study focused on male Chinese individuals, which may not be generalizable to the Chinese population. Second, as a cross-sectional study, the emotion and alcohol dependence status of the participants were only measured once; thus, the long-term association between depression and alcohol dependence among participants with different genotypes could not be detected. Third, the data were collected using self-rating scales, so there may be reporting bias.

In conclusion, there was an interaction between NPY *rs16147:T*>*C* and alcohol dependence indicating that *rs16147:T*>*C* might correlate with susceptibility for depressive symptoms among male adults with alcohol dependence during the period of ADW. These findings provide support for the differential susceptibility model, in which the CC homozygote of *rs16147:T*>*C* was a plasticity factor rather than a factor that only increased the depression symptoms of individuals during ADW. These empirical findings have important implications for understanding the genetic moderation of alcohol dependence and its effect on individual differences in depression symptoms during the period of ADW. Further work is required to explore the underlying mechanisms of depression modulation at the molecular level, especially in relation to functional studies of neural systems.

## Data availability statement

The original contributions presented in the study are included in the article/Supplementary material, further inquiries can be directed to the corresponding authors.

## Ethics statement

The studies involving human participants were reviewed and approved by the Ethics Committee of Peking University Health Science Center. The patients/participants provided their written informed consent to participate in this study.

## Author contributions

XW: designed the study and gathered the data. FC, SZ, JZ, KX, and GS: data curation and formal analysis. HS, FY, LH, and YZ: wrote the original draft. Y-HC and YL: revised the paper and helped with other tasks. LC, FW, and WW: gave guidance and modified the manuscript. All authors have approved it for publication. All authors contributed to the article and approved the submitted version.

## Funding

This work was supported by the Natural Science Foundation of Xinjiang Province [2018D01C239]; the Science and Technology Program of Wenzhou [Y20190098]; the National College Students Innovation and Entrepreneurship Training Program [201910343031]; the Natural Science Foundation of Ningbo [202003N4041].

## Conflict of interest

The authors declare that the research was conducted in the absence of any commercial or financial relationships that could be construed as a potential conflict of interest. The reviewer XL declared a past co-authorship with the author FW to the handling editor.

## Publisher's note

All claims expressed in this article are solely those of the authors and do not necessarily represent those of their affiliated organizations, or those of the publisher, the editors and the reviewers. Any product that may be evaluated in this article, or claim that may be made by its manufacturer, is not guaranteed or endorsed by the publisher.

## Abbreviations

NPY: neuropeptide Y
ADW: alcohol dependence withdrawal
cAMP: AMP
CRF: corticotropin-releasing factor
GxE: gene-by-environment
MAST: Michigan Alcoholism Screening Test
SDS Self-Depression Scale
RoS region of significance.
