# Supplementary material for: The neuropeptide Y single-nucleotide polymorphism rs16147:T>C moderates the effect of alcohol dependence on depression in male Chinese Han population
Source: Front Psychiatry. 2022 Sep 29;13:1012850. doi: 10.3389/fpsyt.2022.1012850 (PMC9558829; doi:10.3389/fpsyt.2022.1012850)
Supplement: Supplementary file 1 [file Data_Sheet_1.docx]

Supplementary Table 1 Genotype Frequencies of NPY rs16147:T>C by Hardy-Weinberg Equilibrium in study subjects

| Genotype | | Number of study subjects | | Percentage | |
| --- | --- | --- | --- | --- | --- |
| CC | | 60 | | 13.19 | |
| CT | | 213 | | 46.81 | |
| TT | | 182 | | 40.00 | |
| χ2 | 0.04 | | p | | 0.85 |

Supplementary Table 2 Genotype distribution of the NPY *rs16147* polymorphism in individuals with alcohol dependence and depressive symptoms

| NPY *rs16147* | Alcohol Dependence | Depression |
| --- | --- | --- |
| CC homozygote, M (SD) | 10.02（5.70） | 56.22（14.60） |
| T allele carriers, M (SD) | 9.16（5.43） | 56.04（10.84） |
| *t* | 1.13 | 0.11 |
| *p* | 0.26 | 0.93 |

Note：NPY= Neuropeptide Y; T allele carriers= CT or TT; M= mean; SD= standard deviation; t= t-value; p= p-value.
